# SPECT/CT Assessment of In-Vivo Loading of the Knee Correlates with Polyethylene Deformation in Retrieved Total Knee Arthroplasty

**DOI:** 10.3390/tomography8010015

**Published:** 2022-01-07

**Authors:** Harry Hothi, Arianna Cerquiglini, Lukas Büel, Johann Henckel, Lukas B. Moser, Michael T. Hirschmann, Alister Hart

**Affiliations:** 1 The Royal National Orthopaedic Hospital, Stanmore HA7 4LP, UK; arianna.cerquiglini.15@ucl.ac.uk (A.C.); j.henckel@ucl.ac.uk (J.H.); a.hart@ucl.ac.uk (A.H.); 2 Department of Orthopaedic Surgery and Traumatology, Kantonsspital Baselland (Bruderholz, Liestal, Laufen), 4101 Bruderholz, Switzerland; lukas.bueel@stud.unibas.ch (L.B.); lukas.moser@donau-uni.ac.at (L.B.M.); michael.hirschmann@unibas.ch (M.T.H.); 3 Department of Orthopaedics, University of Basel, 4001 Basel, Switzerland

**Keywords:** SPECT/CT, TKA, retrieval analysis

## Abstract

Background: SPECT/CT distribution patterns in patients with total knee replacements have previously been correlated with factors such as time of implantation, implant type and alignment. It is unknown, however, if an increased and more extended bone tracer uptake (BTU) in SPECT/CT, representing loading of the joint, correlates with findings from retrieval studies. The aim of this study was to further understand this subject. Materials and Methods: 62 retrieved TKA were included. SPECT/CT was performed prior to revision. Quantitative and qualitative medio-lateral comparisons of BTU intensity and distribution in the tibia were performed. Retrieval analysis was performed with a micro-CT method to assess the thickness differences between medial and lateral sides of polyethylene inserts with symmetrical designs. Results: In the subgroup of TKA with asymmetrical SPECT/CT distribution, there was a significant correlation between retrieval and medical imaging data (*p* = 0.0355): patients showing a more extended BTU in the medial compartment also had a significantly thinner insert in the medial compartment, and vice versa in the lateral side. Conclusion: This is the first study comparing BTU distribution patterns and retrieval findings. Our results support the hypothesis that SPECT/CT is able to identify bone activity due to implant position and loading.

## 1. Introduction

Some patients are not fully satisfied after total knee arthroplasty (TKA) and this is often associated with unexplained pain [1,2,3]; there are multiple factors which may explain why this occurs. It is important that in patients with a painful TKA, the root cause of this is identified so that the optimum treatment strategy can be implemented [4]. Besides a thorough patient history and detailed clinical examination, the current diagnostic process includes radiological and radionucleotide imaging [5], although widely used, conventional radiographs, magnetic resonance imaging (MRI), computed tomography (CT), scintigraphy and single photon emission computed tomography (SPECT) may not provide the entire picture and may not be enough to establish the pain generator. However, several studies have demonstrated the clinical value of hybrid SPECT/CT in painful TKA [3,4,6,7,8,9,10]. SPECT/CT combines information about the osteoblastic metabolism (SPECT) and the anatomical structure (CT), providing a wider view of the mechanical, structural and biological conditions of the joint. Hirschmann et al. were able to correlate SPECT/CT distribution patterns and intensity thresholds with specific problems such as mechanical loosening, instability, component malposition and patella-femoral disorders, in both TKA and uni-compartmental knee arthroplasty [3,7,9,11,12]. It has also been shown that other aspects might influence the bone tracer uptake (BTU) in SPECT/CT. Hirschmann et al. found a significant correlation between coronal alignment and BTU of the medial and lateral compartments in the natural knee joint: the BTU distribution pattern was associated with conditions of overloading [13].

Retrieval analysis can help us further understand in-vivo implant performance and the influence of position and load distribution [14]. The association between increased bone tracer uptake in SPECT/CT, representing increased loading of the joint, and findings from retrieval studies have not, however, previously been investigated. The potential increase in force transmission and greater damage to an implant may not always cause pain; however, a better understanding of this association can help further explain the usefulness of SPECT/CT as a measure of joint loading.

The aim of this study was to gain insight into this subject, by correlating evidence from retrieval analysis and SPECT/CT images pre-revision in TKA: we expected to find areas of increased tracer uptake in relation with the most deformed compartment of the polyethylene (PE) insert.

## 2. Materials and Methods

**Figure 1:**
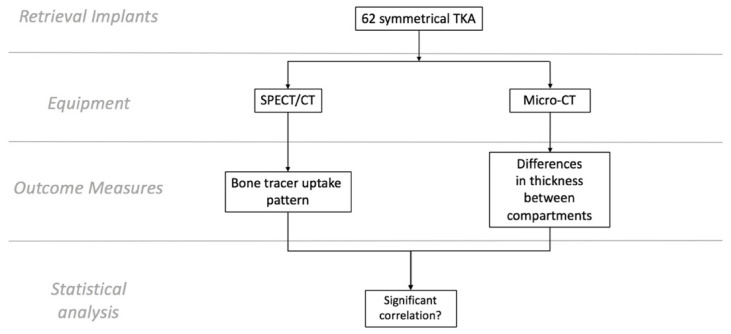
Flowchart showing the study design.

This study involved 62 consecutively retrieved TKAs received at our center. Only implants with symmetrical medio-lateral designs of the polyethylene tibial inserts and for which pre-revision SPECT/CT imaging was performed were included in this study. The retrieved implants consisted of 11 different designs. Figure 1 shows the study design.

All TKA were revised at a single institution by one experienced, high volume knee surgeon. The implants were retrieved from 41 female and 21 male patients, with a median (range) age of 62 (35–84) years. 

The median (range) time to revision was 37 (7–169) months and instability was the most common reason for revision (*n* = 26), followed by malposition (*n* = 16), patellar mal-tracking (*n* = 7), pain (*n* = 7), aseptic loosening (*n* =5) and stiffness (*n* = 1). 

Ethical approval was obtained (07/Q0401/25), and all the patients gave informed consent for participation in the study.

### 2.1. Retrieval Analysis

A micro-CT scanner (XTH 225, Nikon Metrology NV, Derby, UK) was used to image all the polyethylene inserts with a peer-reviewed method [15], setting the X-ray tube voltage to 80 kV and the current to 300 μA, with a resolution of 45 μm. 

From the reconstructed images, iso-surface renderings of each insert were generated using Avizo software (Avizo, FEI Visualization Sciences Group), and thresholding algorithms were applied to optimize geometrical accuracy. The resulting geometry was converted into a stereolithography file format and analysed with Geomagic Control X (Geomagic Inc, Morrisville, NC, USA), in order to identify the thinnest points in both medial and lateral compartments and compute the thickness difference: negative values signify that the medial compartment is thinner than the lateral compartment, and vice versa with positive values. The resulting deformation was considered as a combination of wear and creep; no distinction between wear and creep was made in the present study.

### 2.2. Radiological Imaging Evaluation

99mTc-HDP-single-photon emission computerized tomography (SPECT/CT) [6] was performed using a hybrid system, Symbia T16 (Siemens, Erlangen, Germany), equipped with a pair of low-energy, high-resolution collimators. This system incorporates a dual-head gamma camera with an integrated, 16, 0.75-mm slice thickness CT. All patients received a commercial 700 MBq Tc-99 m HDP injection (CIS Bio International Sur Yvette, Saclay, France). At 2 h after injection, anterior and posterior views of both knees were obtained. SPECT/CT was performed with a matrix size of 128 × 128, an angle step of 32, and a time per frame of 25 s. 

After an interactive reconstruction of the data, images of the tibial components were displayed in the axial plane. Bone tracer uptake (BTU) in SPECT/CT was displayed with a Likert like color coded scale. 

**Figure 2:**
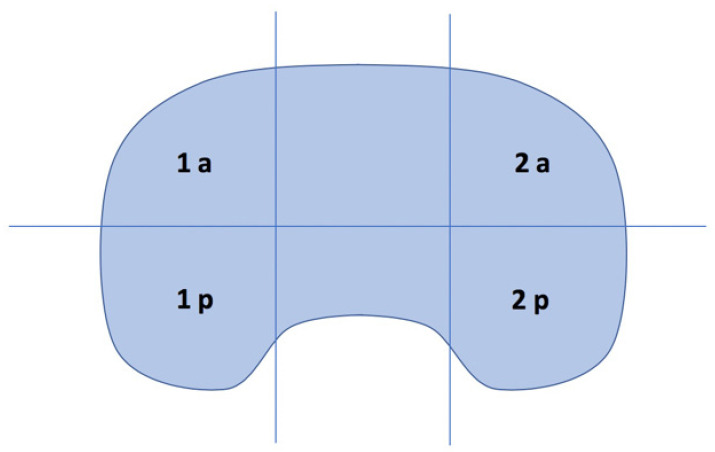
The division scheme of the tibial region for the SPECT/CT localization [10]. In the present study only the medial (1a and 1p) and lateral (2a and 2p) sides were analysed.

The tibial region was divided in 6 zones and mean measurements of BTU intensity were performed, as shown in Figure 2 [9]. A quantitative comparison of BTU intensity between medial (zones 1a and 1p) and lateral (zones 2a and 2p) compartments was made: the maximum BTU intensity value for both medial and lateral compartments was identified, and the difference computed. 

**Figure 3:**
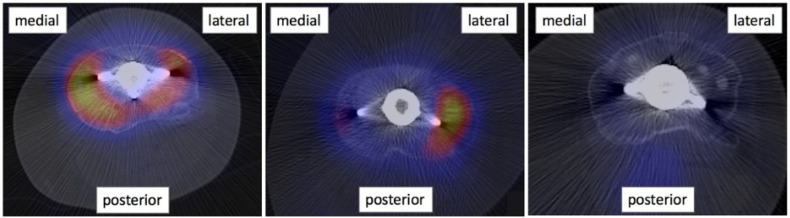
Examples of SPECT/CT images with axial view on the tibial tray. From left: knee with most extended BTU on the medial side of the tibial tray (asymmetrical distribution); knee with most extended BTU on the lateral side of the tibial tray (asymmetrical distribution); no increased BTU detected (symmetrical distribution).

A qualitative comparison of BTU distribution patterns between medial and lateral compartments of the tibial tray was made and the compartment with most extended BTU was noted. The SPECT/CT images were then classified into three groups: the ones showing a higher BTU on the medial side, the ones showing a higher BTU on the lateral side and the ones with no increased BTU or a symmetrical BTU distribution (Figure 3).

### 2.3. Statistical Analysis

Statistical analyses were performed using Prism 6 (GraphPad, San Diego, CA, USA). A post-hoc power analysis was performed. A non-parametric Spearman correlation test was performed to investigate potential correlations between results from retrieval analysis and BTU intensity. 

A *t*-test was performed to compare the micro-CT results between TKAs showing asymmetrical and symmetrical BTU in SPECT/CT. 

Analysing the subgroup of TKA with asymmetrical BTU distribution in SPECT/CT, a Fisher’s test was performed in order to identify a possible correlation between results from retrieval and medical imaging analyses. A t-test was performed to compare the micro-CT results between TKA showing higher BTU in SPECT-CT on medial and lateral compartments. *p* values < 0.05 were considered statistically significant.

## 3. Results

### 3.1. Retrieval Analysis

Micro-CT analysis revealed that 56% (*n* = 35) of tibial inserts showed thinner PE thickness in the medial compartment, with a median value (range) of difference in thickness of −0.075 mm (−3.380 mm–−0.009 mm), whilst 44% (*n* = 27) of tibial inserts showed thinner PE thickness in the lateral compartment, with a median value (range) of difference in thickness of 0.065 mm (0.005 mm–0.600 mm).

### 3.2. Radiological Imaging Evaluation

Mean, standard deviation, maximum and minimum values of BTU intensity are reported in Table 1.

Results from the qualitative SPECT/CT analysis revealed that the medial compartment had a more extended BTU pattern distribution in 48% (*n* = 30) of patients, whilst 27% (*n* = 17) had a more extended BTU pattern distribution on the lateral side. 25% (*n* = 15) of patients had a symmetrical BTU distribution pattern.

### 3.3. Statistical Analysis

A post-hoc power analysis revealed that this correlation study (t test, point biserial model) had a power of 68 (two-sided hypothesis), considering our cohort of 62 samples and assuming a medium effect size (*p* = 0.3).

A non-parametric Spearman correlation and a Fisher’s exact tests revealed that there was no significant correlation between results from retrieval analysis and BTU intensity (*p* = 0.9902 and *p* = 0.5510, respectively).

There was no significant difference in the magnitude of polyethylene deformation between TKAs with asymmetrical and symmetrical SPECT/CT signals (*p* = 0.7418).

**Figure 4:**
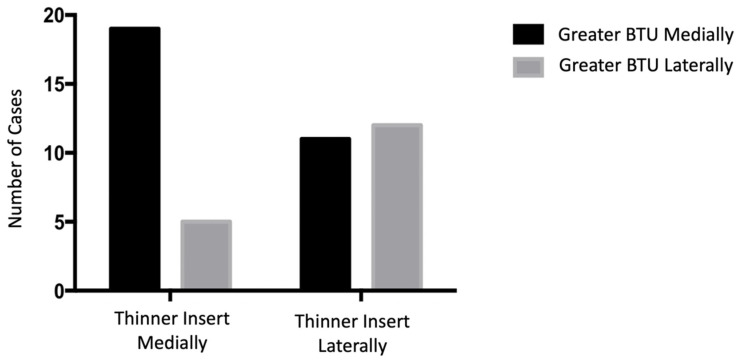
Graph showing the correlation between micro-CT and SPECT/CT data: a more extended BTU on the medial compartment (black boxes) was significantly correlated with a thinner insert in the medial compartment; whilst a more extended BTU on the lateral side (grey boxes) was significantly associated with a thinner insert in the lateral compartment.

**Figure 5:**
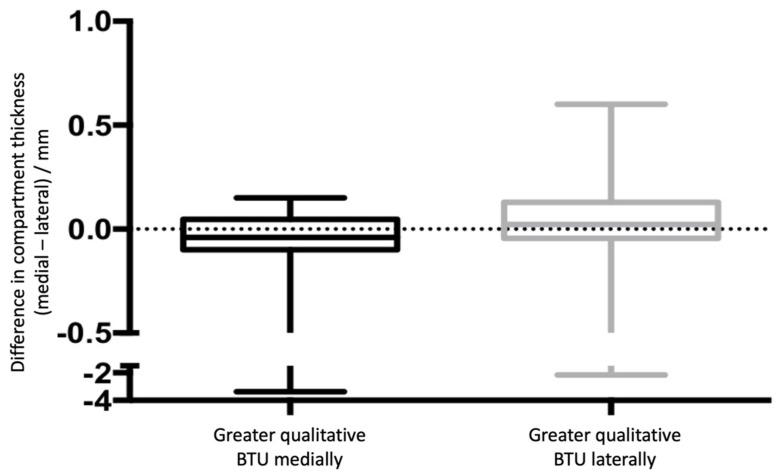
Box plot showing the medial and lateral compartment thickness for the two groups in which either the medial or lateral side was found to have a greater BTU from analysis of SPECT/CT imaging. A negative value on the y-axis indicates that the medial compartment was thinner; a positive value indicates that the lateral compartment was thinner.

**Figure 6:**
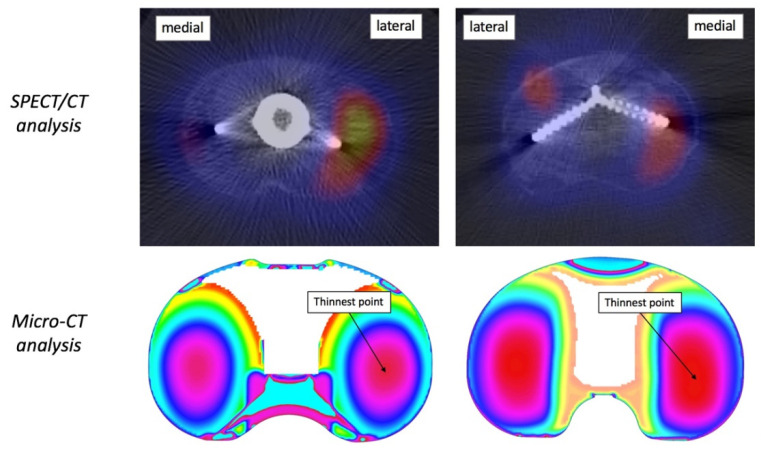
Examples of correlation between SPECT/CT and micro-CT data. (**Left**) left knee showing a more extended BTU on the lateral side, which correlates with a thinner lateral compartment; (**Right**) right knee showing a more extended BTU on the medial side, which correlates with a thinner medial compartment.

Analysing the subgroup of TKA with asymmetrical SPECT/CT signal (*n* = 47), there was a significant correlation between retrieval and medical imaging data (*p* = 0.0355). Patients showing a more extended BTU in the medial compartment also had a significantly thinner medial compartment. Similarly, patients showing more extended BTU in the lateral compartment also had a significantly thinner lateral compartment (Figure 4). Figure 5 presents a box plot of the medial/lateral thickness differences between the two groups where there was greater BTU qualitatively medially and laterally. Examples of this correlation are shown in Figure 6.

## 4. Discussion

This is the first retrieval study correlating evidence from retrieval analysis with pre-revision SPECT/CT in patients with a painful knee after TKA. Our most important finding was a correlation between PE insert medial/lateral thickness changes and qualitative SPECT/CT distribution patterns.

Previous studies have investigated the association between TKA position and BTU patterns in SPECT/CT. In a prospective study with 100 TKA patients [3], Hirschmann et al. identified different SPECT/CT distribution patterns that correlated with clinical findings: in particular, it was reported that increased BTU in the medial-lateral regions was associated with a varus/valgus position of the tibial component. The same research group reported a clear correlation between the alignment of the natural knee in the coronal plane and the intensity of BTU in the medial and lateral compartments [13]. Unbalanced force distribution in the natural or replaced knee joint can lead to overloading and bone re-modelling in specific areas, where it is possible to observe an increased BTU [3]. Computational [15] and cadaveric [16] studies have explained how conditions of malposition in the coronal plane can affect load distribution and increase contact stresses in particular areas of the bone.

In parallel, several retrieval studies have demonstrated the correlation between implant position and deformation of the polyethylene tibial component [14,17,18,19]: varus alignments were associated with more deformed medial compartments, whereas valgus alignments were associated with more deformed lateral compartments. Our present study helps to join up these previous clinical and retrieval studies by demonstrating that the compartment with the more extended SPECT/CT signal showed the comparatively thinnest PE thickness. These findings add to the important discussion of the utility of SPECT/CT imaging in identifying differences in force transmission and loading between the medial and lateral compartments of the knee. Indeed, it has previously been shown that varus/valgus alignment can increase the risk of the progression of osteoarthritis in the compartment that consequently experiences greater loading due to the alignment [20]. These early changes in the joint (i.e., during the first 1.5 years) are often difficult to detect on plain radiographs alone; that is to say that it is challenging for radiographic analysis to identify the early impact of overloading of one compartment. SPECT/CT imaging can present useful data pointing towards early signs of compartment overloading in the knee; our study shows this to be case at the broader qualitative level at least. 

No difference in the extent of deformations were found in implants with symmetrical and asymmetrical BTU distribution patterns and data from quantitative analysis of the BTU intensity did not correlate directly with retrieval findings. The absence of a correlation with quantitative BTU data may be explained by the presence of other potentially influencing factors, including conditions of tibial loosening, instability or malposition in the axial and sagittal planes and any differences in fixation and implant design; all these may impact the changes in the BTU assessed [3,7,9,11,12,21]. It is also possible that factors directly related to the patient’s anatomy play a role in the loading distribution; Slevin et al. speculated that alignment target based on the individual knee morphotype should be taken in consideration to improve clinical outcome [22].

The present study has some limitations, which are related to the limitations of all retrieval studies. Firstly, the number of implants included in the study is comparatively low. However, the use of hybrid SPECT/CT imaging in the routine diagnostic process remains low, therefore recruiting more implants with this imaging data is challenging. SPECT/CT is not a standard test performed in the case of pain after a TKA, partly due to the associated cost of the scan which can be twice that of performing a CT scan [10].

Secondly, our cohort consisted of 10 different cemented and uncemented implant designs, with several different reason for revision: more control on these variables is essential in order to better identify and differentiate different BTU distribution patterns in SPECT/CT and their etiology. Future studies should also compare findings with those from patients with well-functioning TKAs.

Lastly, it is noted that in some cases, the differences in measured thickness between the medial and lateral sides was comparatively very small, and close to or at the limit of what can be accurately measured with micro-CT reconstruction. In this study, however, the primary focus was to determine the relative differences between medial and lateral compartments rather than on absolute values. Future investigations focusing on absolute values of thickness should control for this accuracy variable in their study design. 

## 5. Conclusions

This study is the first to compare pre-revision SPECT/CT data and micro-CT based retrieval data, from patients with painful TKAs. Our key finding was that the location (medial vs lateral) of BTU patterns significantly correlates with polyethylene deformation in tibial inserts; this may help predict load distribution in vivo. This study provides useful information that support previous findings on the utility of hybrid SPECT/CT imaging in the routine diagnostic process.

## Figures and Tables

**Table 1:** Mean, SD, minimum and maximum values of BTU intensity for each of the 4 tibial zones analysed.

	Mean	SD	Minimum	Maximum
1a	1.48	1.02	0.52	5.68
1p	1.76	0.90	0.55	5.23
2a	1.53	0.92	0.61	4.47
2p	1.74	0.92	0.79	4.92

## Data Availability

Not applicable.
